# The Life Cycle of the Root Borer, *Oryctes agamemnon*, Under Laboratory Conditions

**DOI:** 10.1673/031.008.6101

**Published:** 2008-10-23

**Authors:** Rasmi Soltani, Ikbel Chaieb, Med Habib Ben Hamouda

**Affiliations:** Biological Sciences and Plant Protection Department, Agronomics Sciences Institute of Chott Meriam 4042-Sousse, Tunisia

**Keywords:** date palm

## Abstract

The root borer, *Oryctes agamemnon* Burmeister (Coleoptera: Scarabaeidae), has become a serious pest of date palm trees in southwest Tunisia. Under natural conditions, mated females lay eggs in different parts of palm tree: between the hairy roots, all along the stem at the leaf axils and at the base of cut branches. Larvae bore into targeted places of the plant and were never seen outside. Pupation takes place in the plant and emergence of the adults begins in June. Larval feeding causes extensive damage to the respiratory roots. To examine the life cycle more closely, the *O. agamemnon* life cycle was studied under laboratory conditions. Different larval stages were collected from infested oases in Tozeur and placed in plastic boxes with natural food that was collected from the oases. After emergence, adults were paired in opaque plastic boxes for mating with the same food substrate which also served as an oviposition site. Eggs were collected daily and isolated in new boxes. Hatched eggs were recorded. The number of larval instars was determined by measuring the width of cephalic capsules. Under laboratory conditions (23 ± 2'C and 55 ± 6% RH)embryogenesis took 14.3 ± 1.42 days and the first, second and third larval instars were 33.1 ± 2.69, 63.88 ± 6.6 and 118.3 ± 13.38 days respectively. The pupal period lasted 24.1 ± 3.02 days and the adult 65.27 ± 9.48 days. These facts indicated that *O. agamemnon* is univoltine.

## Introduction

The genus *Oryctes* includes 39 species (Bedford 1976), but only some of them impact the development of palm trees and more particularly coconut trees (Ohler 1999). Due to its wide distribution, the species *Oryctes rhinoceros* (L.) is the most important and studied pest of coconut (Gressit 1953; Ohler 1999). Its attack kills both seedlings, young and old palms and can discourage replanting of coconut (Sivapragasam 2003).

The root borer (Balashowsky, 1962) *Oryctes agamemnon* Burmeister (Coleoptera: Scarabaeidae) was introduced accidentally into the oases of Mrah Lahouar in the Djerid zone southwest of Tunisia at the end of 1970s and beginning 1980s from the United Arab Emirates (Anonymous, 2000). It is a pest of various palm trees (Surany 1960 cited in Balashowsky 1962). It was discovered in 1995 by the Phoenicicole Research Centre of Dagache Tunisia, when serious damage caused by this pest began to cause the collapse of certain old and highly productive date palms in the Djerid zone (Khoualdia et al., 1997). It was previously not an important destructive pest of date palm in Tunisia, but it now causes several problems especially in young plantations and the death of offshoots can reach 100% in some cases (Soltani, 2004).

In the field, larvae are responsible for damaging different parts of the date palm and adults do not cause damage. Larvae were localized in both parts of the respiratory roots; the aerial part above the soil and the underground part that reaches a depth of 25 cm Larvae were also found all along the stem in the axils of dry cut down palm, between the leaves, and on the upper surface of green palm on the crown (Linnae 1973 cited in F.A.O 1999).

Larvae and adults are never seen inside the stem. The most sensitive attacked part was the respiratory roots that support the entire mass of the palm and fix it to the soil. Feeding larvae reduce the attacked parts to powder, which resembles to mature compost (Soltani, 2004). Oases are seriously damaged and determining the number of larvae on palm trees is very important. As no effective chemical or biological treatment is available, understanding the life cycle and behaviour of insect pests is a first step to the development of better techniques for their control.

## Materials and Methods

### Collection of samples

Biological material was collected by hand picking, from November 2002 to March 2003, in the oases of Mrah Lahouar and Ibn Chabatt sites in Tozeur, in southwestern Tunisia where palm trees were seriously infested by this pest. Samples included all life stages that provided the initial material for breeding trials.

### Rearing methods

As methods for rearing *O. agamemnon* in the laboratory had not been developed, the first goal was to developing such methods. Different food substrates were tried under laboratory conditions including cauliflower, potatoes and natural substrate collected from the natural breeding sites of larvae. The two first foods were used to test the ability of this species to live on substrates other than the natural one.

Rearing boxes were kept under ambient temperature conditions of 23 ± 2°C. The relative humidity within the boxes was 55 ± 6 %. Holes in the cover of each box decreased the humidity inside.

### Larval period

During the larval period the width of cephalic capsules were measured, using a numeric slide gauge, to determine the number of larval stadia. This operation was done three times per stadium (n = 12 larvae for each stage) as follows: just after moulting and 15 days and 30 days later to verify the change in the dimensions of the cephalic capsule.

Larvae were placed by stadium in groups of three inside opaque plastic boxes. Cauliflower and potatoe tubercles were changed when necessary. Natural food, composed of decayed substrate and fibrous roots, was renewed every 10 days and when necessary if the number of larvae exceeded three per box.

The length of larval stadia began after the moulting of the collected biological material which was mainly composed of first and second instars. Second generation insects were used to determine the length of earlier stages.

The amount of cannibalism was mainly determined by grouping 6 to 8 larvae of the same instar in boxes provided at the beginning of the experiments with normal quantity of food but insufficient quantities thereafter. A total of ten boxes were used during this experiment divided as follow: three boxes with first and second instars and four boxes with the third instar.

Other observations were made by grouping different combinations of stadia, inside the same box, as: i) different larval instars; ii) third larval instar, prepupa and pupa; iii) third larval instar and adults, and iv) third larval instar and eggs.

### Prepupal and pupal period

The prepupal period characterized the end of the third larval instar; it was mainly marked by the static form of larvae and the arrest of feeding activity. The prepupal period ended with pupation.

The number of days elapsed between the pupal stage and adult emergence was considered as the pupal period.

Other measurements of length and width were made on a sample of 20 individuals to characterize larvae.

### Feeding, mating and egg embryogenesis

Adult feeding activity was examined in boxes containing either natural substrate or other foods (cauliflower or potatoes tubercles).

The emerged laboratory adults were placed in pairs into plastic boxes with natural substrate. The mating time was measured and behaviour was observed for seven couples. Laid eggs were daily counted and removed to other boxes containing the same natural decayed substrate with a measured relative humidity of 55 ± 6 %. Eggs were maintained at the surface of the substrate which made it possible to examine them daily examination. The embryonic period, the number of days elapsed between oviposition and hatching of an egg, was measured in a sample of 55 eggs. The length and width of ten eggs was measured.

### Fecundity, female reproductive success and longevity

Pupae developed from experimental material or collected from oases were sexed using the curved horn, which was present on male heads and rudimentary or absent in females.

The number of twenty-eight males and females were paired under laboratory conditions (23°c, RH = 55 ± 5%). Fecundity was measured as the total number of eggs laid per female and female reproductive success was the number of hatched eggs per female. These two parameters and longevity were determined using a sample of 11 to 15 mated females. Longevity was also measured for 10 non-mated females.

### Statistical analysis

Data obtained from experimental insects were subject to one way analysis of variance (ANOVA) with repeated measures. A computer programme, SPSS 13.0 Software, was used to compare the means of various treatments.

## Results and Discussion

### Embryogenesis

Eggs were ovoid in shape, pearly white and measured on average 3.11 ± 0.13 mm in length and 2.25±0.18 mm in width. At the end of embryogenesis, this ovoid shape became nearly round and volume increased 3 or 4 fold, and some parts of the head capsules were visible through the transparent membrane of the egg. Incubation lasted 14.3 ± 1.42 days. This result was similar to those of Lepesme 1947 (cited in Khoualdia O. et al. 1997), which indicated an average period of 13 days for *O. agamemnon .* However, the embryogenic period for *Oryctes rhinoceros* lasted between 8–12 days (Bedford 1976; Sivapragasam 2003).

Mortality during embryogenesis was 9.09 % due essentially to low humidity that results in the arrest of embryonic development, or excess humidity that results in fungal infestation.

### Larval period and cannibalism

Cephalic capsule measurements at different times during each larval stadium remained constant and was used as a criterion to determine the accurate number of larval instars. Results of these measurements showed the existence of three larval instars (Table 1). The growth rate of cephalic capsules was 1.9 from first to second instars, and 1.73 from the second to third instars.

**Table 1.**
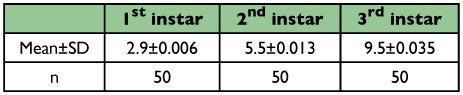
Width of cephalic capsule (mm)

After hatching of the egg and at each moult larvae were characterized by very large cephalic capsules compared to the body and the cephalic capsule was soft with unusable mouth parts. Hardening and chitinisation of the head took 4 hours during which time the larvae remained motionless.

After eclosion, larvae consumed the exuviate of the previous larval instar which constituted the first food consumed by the newly emerged larva.

The length of the body of 1st, 2nd and 3rd instars reached a maximum of 29 ± 0.005, 55 ± 0.014 and 90 ± 0.035 mm, respectively. The most destructive stage is the third instar that can reach 17 g in weight at full development.

Larvae bred on cauliflower died before they molted, but larvae fed on potato tubercles and natural food completed normal development.

The duration of developmental periods of various stages of *O. agamemnon* reared on natural material at 23±2°C (RH=55±6%) are shown in Table 2. The time necessary for all larval instars was a total of 215.3 days with a marked dominance of the third instar that occupied nearly the half of this duration. Lepesme (1947) studied the biology of *O. agamemnon* and found that the duration of the first, second and third instars were respectively, 30–35, 30–45 and 55–95 days, which are shorter for the second and third instar. These differences can be explained by the breeding conditions and the quality of food used. Comparing these results with *O. rhinoceros* (cited by Waterhouse et *al.* 1987) larval durations of 10–12, 12–21 and 60–165 days, respectively for first, second and third instars were reported.

**Table 2.**
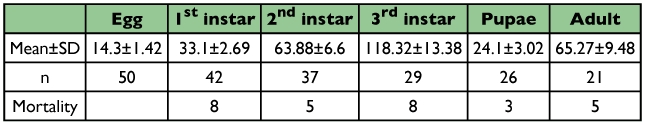
Duration of development of instars (days)

The mortality of larvae fed natural food is shown in Table 3. Common to all stadia was the time to larval mortality that was mainly observed at the beginning of each instar. At this phase, newly eclosed larvae were vulnerable to higher humidity and temperature that may negatively affect feeding. The second factor leading to mortality was caused by cannibalism by larvae.

**Table 3.**
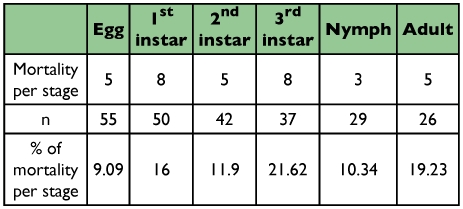
Mortality percentage in different stages

Cannibalism was more accentuated when third larval instar, prepupa and pupa were present together inside the same box. The third larval instar killed and consumed the prepuae and pupae which were static, vulnerable and without protection. When larvae were caged together with adults legs constituted the mainly target of larvae, but their attacks were generally without effect because of the rigidity of the adult body and their mobility which allowed them to easily escape. Occasionally larvae could cut adult legs. No observed cannibalism was seen toward eggs.

The mortality of larvae due to cannibalism in the first, second and third stages was 8.33%, 36.36% and 60.71%, respectively (Table 4). These differences in percentage can be explained by the duration and the voracity of each larval instar. Since these two parameters increased proportionally with larval instar suggests that cannibalism was due to food lack and overpopulation inside boxes. It is not known if cannibalism occurs under natural conditions.

**Table 4.**
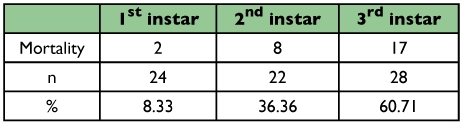
Cannibalism Mortality percentage in different stages

### Prepupal and pupal period

The prepupal period lasted 17.14 ± 3.29 days, but in Table 2 the prepupal stage was included with the third larval stage. The prepupal stage was described by Balachowsky (1962) as having a duration of 8–13 days for the Scarabaeidae: Melolonthinae. It was also mentioned by Bedford (1980).

After moulting the pupa was initially creamy white. This colour persisted for 2 to 3 hours, after which it changes progressively to yellow, yellowish brown, and faint brown after 7 to 8 days. Length, width and weight measurements are shown in Table 5.

**Table 5.**
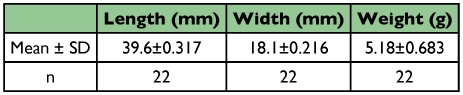
Nymphs dimensions and weight

Both elytra and fully developed hind wings were present, elytra directed nearly laterally to the body were slightly bent under the body. Initially, legs were slightly glued to the body, but they were progressively liberated and sclerified.

The coloration process was initiated at the second period of this stage during which the legs, prothorax and head acquired their final coloration and were also completely sclerified. The abdomen and elytra remained soft until adult eclosion.

The developmental period of the pupa lasted 24.1 ± 3.02 days and ended with imaginal eclosion. These results agreed with those cited by Lepesme (1947), indicating that the pupal stage lasted 20–28 days for the same species. Waterhouse et al. (1987) reported similar results for *O. rhinoceros* (17–28 days).

Pupal mortality shown in Table 3 was mainly caused by: failure of the larval cuticle to detach at the cephalic capsule, burying of the pupa in the substratum after pupal moulting, and irritation or injury resulting in the death of the pupa

Generally, the differences in durations found with those cited by Lepesme (1947) for *O. agamemnon* can be explained by several factors including the conditions of breeding such as the temperature, to the quality of food and its availability. These two parameters can enormously influence the development cycle, prolonging or decreasing it. In fact, under natural conditions, the life cycle of Scarabaeidae varies with climate and is longest in more temperate regions and shorter in tropical areas with no climatic seasons (Ritcher 1957).

### Mating, fecundity, fertility and longevity

Adults showed different feeding behaviours depending on the food offered. They fed in potatoes tubercles by extracting the juice of chewed tissues, adult dig a hole in the potato and stay inside. However, no feeding activity was observed in presence of cauliflower and natural substrate.

Using natural substrate, after its emergence and the end of the process of hardening and maturation adults remained sedentary, buried in the substrate. Adults were never seen to feed during their life time. They apparently live on reserves accumulated during the larval period Adult dimensions and weight are shown in Table 6.

**Table 6.**
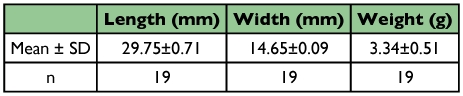
Adults dimensions and weight

Within boxes, first step of mating occurred at the surface of substrate. The male mounts the female by approaching from behind. It straightens its forelegs elevating the anterior part of its body and tries to induce the female to mate by using his tarsi to tap rhythmically on the body of the female. Duration of this act was variable and ended with mating. When receptive the female opened the pygidium and everted the tip of her genitalia allowing the male to insert the aedeagus. While mating the male body was maintained in a vertical position relative to the female. Once copulated the female, supporting the male on her back, dug into the substrate where mating was completed and oviposition occurred. Copulation was observed to occur during the day except during the early morning.

Copulation time, measured for 7 pairs, lasted 62.86 ± 7.82 minutes with duration varying from 54 to 75 minutes. The end of mating occurred when the female pushed the male off using her posterior legs. Female oviposition activity began one to two days after copulation.

The number of eggs laid by female varied between 17 and 31 eggs/female, the mean for seven studied females was 22.57 ± 4.65 eggs.

Oviposition activity is irregular with periods of rest. Egg hatching varied between 0 and 100%. It was affected by humidity inside boxes. The best relative humidity was between 20 and 30%. The average longevity of adults under laboratory conditions was 65.27 ± 9.48 days without considering the sex, and varied from 51 to 82 days.

Mean fecundity of *O. agamemnon* obtained by Lepesme (1947) was 30 eggs/female, compared with *O. rhinoceros* which varied between 30–40 eggs/female (Waterhouse et al. 1987). This large difference was principally due to the heterogeneity of the chosen sample. In laboratory breeding, mating females were heterogeneous in body size that influenced the numbers of laid eggs. The smallest female in body size laid 17 eggs and normal females laid 25 to 31 eggs. So, the number of laid eggs increased proportionally with female dimensions. The size of sample can also influence the results.

In conclusion, determination of an adequate rearing method permitted us to determine the length of every life stage of *O. agamemnon. O. agamemnon* has one generation per year, lasting about 336 ± 10 days when breeding in natural substrate at 23±2°C and 55±6% RH. Thus *O. agamemnon* is a univoltine species. After egg hatching, larvae became established in the substrate and developed as far as the pupal stage. The third stage larval period is quite long, requiring more than 100 days. Adults do not feed. Mating and oviposition occur in dark places inside the substrate.

These laboratory tests constitute a first step to understand the biology and the behavior of the species, but a study of *O. agamemnon* under natural conditions is necessary.
